# Radial arterial waves for chemotherapy- and radiotherapy-related myocardial damage identification in patients with breast cancer

**DOI:** 10.37796/2211-8039.1390

**Published:** 2023-06-01

**Authors:** Chia-Ying Lee, Daniela Yunchin Yen, Mark C. Hou, Ying-Ling Chen, Rong-Jen Shiau

**Affiliations:** a Department of Chinese Medicine, Changhua Christian Hospital, NO. 135 Nanhsiao St., Changhua, 50006, Taiwan; b Department of Post Baccalaureate Chinese Medicine, China Medical University, No. 91 Xueshi Road, North District, Taichung, 404, Taiwan; c Department of Beauty Science and Graduate Institute of Beauty Science, Technology, Chien-kuo Technology University, No.1, Chiehshou North Road, Changhua, 500, Taiwan; d School of Chinese Medicine, China Medical University, No. 91 Xueshi Road, North District, Taichung, 404, Taiwan; e Department of Chinese Medicine, China Medical University Hospital, No. 2 Yude Road, North District, Taichung, 404332, Taiwan

**Keywords:** ***Keywords:*** Breast cancer, Chemotherapy, Radial pulse wave, Radiotherapy

## Abstract

**Introduction:**

Chemotherapy and radiation therapy for breast cancer cause side effects, such as cardiovascular changes, which can be monitored with echocardiography. However, more convenient methods are always encouraged. Radial arterial waves that are used to detect cardiovascular changes can be used to assist in confirming cardiovascular changes.

**Aim:**

This retrospective study aimed to analyze the frequency and time domains of the radial artery pulse wave in patients with breast cancer to understand its effectiveness in identifying cardiovascular changes.

**Methods:**

Patients with breast cancer were screened from the pulse examination records in Changhua Christian Hospital and divided into the treatment and remission groups. After unlinking the data, the pulse data were analyzed for the breast cancer treatment and remission group, including the average value of the parameters of four consecutive pulse diagnosis records in four consecutive months to test the difference in pulse waves due to breast cancer treatment between the two groups. Additionally, the pulse wave stability of the two groups was compared using the coefficient of variation.

**Results and conclusion:**

The comparison of the pulse wave data between 19 patients in the treatment group and 40 patients in the remission group revealed 45 parameters in time and 50 in frequency domains. D3, ND3, NA1, and NT1 are the four parameters with significant differences (p < 0.05), which are all related to heart function, and mainly related to cardiac output and peripheral resistance, indicating that patients in the treatment period have poor heart function. No difference was found in the degree of data dispersion between the two groups. Cardiovascular side effects caused by breast cancer treatment can mainly be shown in the pulse wave time domain.

## 1. Introduction

Breast cancer is a malignant tumor with the highest incidence among females [1]. Patients with breast cancer of various stages are often treated with radiotherapy and chemotherapy in addition to surgical treatment. Both radiotherapy and chemotherapy may have adverse effects and damage normal cells, such as vomiting, hair loss, oral ulcers, pancytopenia, etc., which will affect the quality of life of patients in the long run [2,3].

Among the side effects of chemotherapy drugs for breast cancer, anthracyclines (such as doxorubicin), taxanes, fluorouracil, cisplatin, and cyclophosphamide are known to cause heart disease [4,5,6]. Monoclonal antibodies, such as trastuzumab and bevacizumab, have also been analyzed for the relationship between heart failure, arrhythmia, and hypertension after treatment [7,8]. Radiation therapy mainly causes blood vessel damage and has a little myocardial effect [9]. The blood flow to the heart will be affected when the myocardial capillaries or coronary arteries are damaged. Thus, experts have begun to pay attention to its prevention and management due to the possible acute and chronic symptoms of cardiotoxicity caused by radiotherapy and chemotherapy, and even life-threatening for patients. Toxicity-related diagnostic and treatment guidelines have been proposed [10,11]. Electrocardiograms and echocardiography were mainly used to detect cardiovascular damage and further necessary procedures were conducted [12]. Myocardial damage caused by breast cancer treatment has been confirmed and relevant guidelines are presented as the basis for evaluation and treatment.

More and more patients with cancer choose alternative medicine as adjuvant therapy during chemotherapy and radiotherapy of western medicine; thus, they seek the help of traditional Chinese medicine (TCM) to reduce the side effects caused by cancer treatment [13,14]. Pulse diagnosis is a unique diagnosis method in TCM. The doctor places the fingertips on the radial side of the patient’s wrist, which reflects the vibration amplitude, frequency, and depth of different radial arteries, due to the patient’s condition, which can be used to accordingly diagnose and give treatment [15,16,17]. Today, TCM practitioners have begun to use equipment to detect radial artery waves, convert radial artery information into waveforms, and conduct data analysis to increase the diagnosis accuracy [18,19]. ANSWatch (TS-0411 Science Co., Taiwan) is non-invasive and can measure radial artery waves in real-time. The operating principle is the oscillometric method, which records the pressure wave of the radial artery. The radial artery wave is analyzed in time and frequency domains to understand the current physiological condition of the subject [20,21]. ANSWatch has been widely used in many studies with comparable results, such as the cardiovascular effects of stimulant beverages [22], and studying the relationship between driving concentration and pulse wave [23,24]. Cardiovascular-related problems caused by diabetes are also observed using pulse waves [25]. Compared with echocardiography, pulse wave examination is convenient and inexpensive and can be used to detect cardiovascular changes.

This retrospective study aimed to analyze the radial pulse waves of patients with breast cancer during the treatment and remission period after treatment completion, and compare the differences in cardiovascular damage during chemo/radiotherapy and after treatment from radial pulse waves.

## 2. Materials and methods

### 2.1. Sample collection

**Fig. 1:**
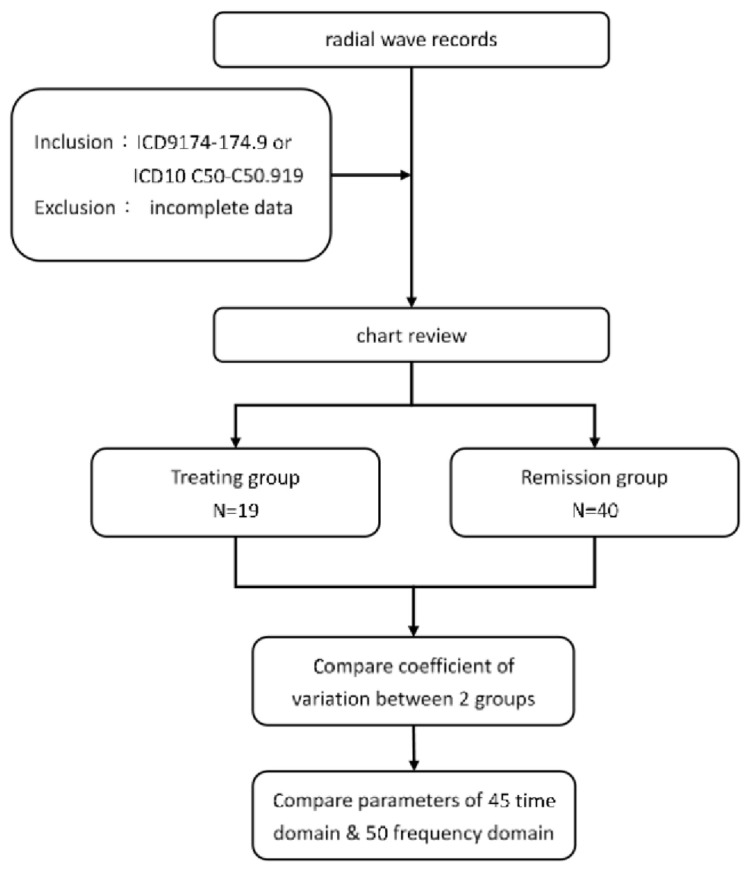
Flowchart of the study.

This retrospective study reviewed the pulse examination data of patients who were diagnosed with breast cancer and had undergone pulse examination once a month for four consecutive months in the TCM Department of Changhua Christian Hospital. Inclusion criteria were patients diagnosed with breast cancer between 2012 and 2019, with the International Classification of Diseases (ICD) 9 (174–174.9) or 10 (C50–C50.919). The exclusion conditions were those with incomplete basic or pulse data. Participants were divided into two groups through chart review. Regular treatment (including surgery, chemotherapy, radiotherapy, and targeted therapy) was received by 40 patients that consisted of the treatment group. Another 40 patients have had treatment and consisted the remission group (excluding hormone therapy). After comparing the dispersion of the two groups with the coefficient of variation, the pulse wave of the two groups was statistically analyzed (Fig. 1).

### 2.2. Data characteristics

This study used ANSWatch to measure and record the radial artery wave. The pressure sensor is placed on the radial styloid process of the wrist to measure the blood pressure wave signal of the radial artery. The operating principle of the instrument is the oscillometric method. The feature point parameters in the pulse wave image are the result of the combined action of the heartbeat and the surrounding tissue of the blood vessel [26].

The AnsWatch embeds a contact piezoelectric sensor in the wristband. This biosensor contains multiple piezoelectric elements, no matter the size of the hand, it can be aimed at the radial artery of the hand to measure accurate pulse waveforms. The position of the piezoelectric sensor is based on the ergonomic design of the left hand, so the test can only be performed on the left hand; the AnsWatch first measures the blood pressure of the subject, and obtains the systolic blood pressure, diastolic blood pressure and mean arterial pressure. Then, based on the subject’s systolic blood pressure, different pressures (20 mmHg decreasing) were applied to measure the “sink”, “middle” and “floating” radial artery blood pressure waves. Finally, the three different waves were measured as Time domain analysis of the subject’s pulse. The time domain data is transformed into the frequency domain data by Fourier transform. The samples were bent every 5 s for a total of 7 min.

The radial artery blood pressure wave is analyzed in time and frequency domains to understand the current physiological condition of the participants. Therefore, cardiovascular-related research is often conducted using pulse diagnostic instruments [27].

#### 2.2.1. Time domain analysis

Radial pulse wave has several physiological information, including myocardial systolic blood output, aortic valve opening and closing, mitral valve atresia, peripheral vascular resistance, etc. [20]. The radial pulse wave is analyzed by time, peak number, main wave (peak, trough), secondary wave (peak, trough), time parameter, pressure parameter, oblique angle parameter, and area parameter to analyze the physiological meaning of radial artery blood pressure wave. The time domain analysis obtained 45 parameters. The main parameters are outlined below. The first peak is the P-wave, which is the largest amplitude wave caused by a large amount of blood released by the ventricles when the heart contracts. P-wave amplitude is the main indicator reflecting vascular filling degree or pulsatile cardiac blood supply. The second peak is the D-wave, which is a prominent wave in the descending branch of the P-wave. The aortic valve is generally believed to suddenly close in the early ventricular diastole, and the blood backflow hits the aortic valve and rebounds the blood flow, making the aortic pressure slightly rise again, and the aortic elastic recoil wave is formed by the slight blood vessel expansion. The first wave trough is point A, which is the pressure and volume in the blood vessels at the end of the diastole. The second wave trough is the point U, which is the starting point of the main wave trough, the ejection time of the aortic opening, which represents the rapid blood ejection from the ventricle, the starting point of blood flow into the artery, and the so-called inflow point. The third wave trough is the point V, which is the downward wave formed by the descending branch of the P-wave and the ascending branch of the D-wave. It represents the emptying time of aortic static pressure, which is the dividing point between systole and diastole. The first angle is the P angle, which is the angle between the ascending and descending branches of the P-wave. A large angle within a limited range indicates better vascular function. The U–P time is T1, which is the time it takes for the inflow point to rise to the main peak. It reflects the time when the left ventricle begins to contract and the arteries dilate, rapidly ejecting blood to the maximum output. The P–V time is T2, which is the time required for the main wave to drop to the inflow point, reflecting the speed of cardiac output retraction. P–V time is the late ventricular ejection and slow ejection time. V–D time is the ventricular diastole and aortic valve closure time. A–A′ or U–U′ time is one pulse cycle time. The U–P slope is ND1, which is the inflow point that rises to the main peak rise velocity and is affected by cardiac output, arterial resistance, and wall elasticity.

#### 2.2.2. Frequency domain analysis

The radial pulse wave is transformed by Fourier transformation, and the frequency domain spectrum can be analyzed, including the sum of a constant (fundamental frequency) and a set of sine and cosine functions with a common period (octave). Wang divided the radial pulse wave into 0 to 12 resonances and explained the organs according to the motion frequencies of different organs observed in the animal experiments, and different frequencies correspond to different organs [28]. In this study, the frequency domain analysis obtained 50 parameters. The main parameters are outlined below. Harmonic normalized (%) represents the internal organs. The percentage of 0-, 1-, 2-, 3-, 4-, 5-, 6-, 7-, 8-, 9-, 10-, and 11-octave to full octave represents the pericardial, liver, kidney, spleen, lung, stomach, gallbladder, bladder, large intestine, Sanjiao, small intestine, and small intestine meridians, respectively. These percentages represent the heart meridian [29].

### 2.3. Statistical methods

#### 2.3.1. Coefficient of variation comparison

The coefficient of variation can be obtained by dividing the average value and the standard deviation of the four consecutive data of pulse wave time domain and frequency domain parameters, which can represent the data dispersion degree [30].

The cv of each parameter was obtained by taking the average value of four consecutive data for each parameter and dividing the average value of all numbers of the group by the standard deviation. Statistical differences between the two groups in time and frequency domains were compared using the *t*-test [31].

#### 2.3.2. Comparison of radial pulse wave parameters

The four consecutive pulse wave data of the two groups were averaged, and all parameters of the two groups were tested using the t-test to find out the differences. The results were analyzed and interpreted using the physiological meanings represented by the parameters.

3.3 This study uses R software for statistical analysis. The relevant calculations are placed at https://drive.google.com/drive/folders/1dpXMtb9x5v9BDAbYie6GLYRXzPwe1aWe?usp=sharing for inspection. This study was approved by the Institutional Review Board of Changhua Christian Hospital (No: 190,502).

## 3. Results

In this study, a total of 40 breast cancer pulse wave data were collected in the remission group and 19 in the treatment group after deducting inconsistent or incomplete data. All are females. The average age of the treatment group was 49.11 ± 7.15 years, and the average age of the remission group was 52.88 ± 9.26 years.

Each patient has four monthly records of pulse data (Table 1).

The coefficient of variation between the two groups did not reach a statistical difference, indicating that the pulse wave data of the two groups were similar in degree of dispersion in both time and frequency domains (Table 2).

The time domain obtained 45 parameters and the frequency domain obtained 50 parameters in the remission group and the treatment group respectively. D3 (DC/d-c), ND3 (Dd-Cc)/(cd/ag), NA1 (A1/A10), and NT1 (b-a/g-a) are the four significantly different parameters between the two groups (*p* < 0.05), the rest are not significantly different. The four parameters were all related to cardiac function, indicating a poor cardiac function of patients during the treatment period (Table 3).

NT1 represents the rising slope of the pulse wave, which has proportional physiological significance to left ventricular contractility, cardiac output, and systolic blood pressure. D3 is the rising slope of the second wave (diploic wave), which is physiologically proportional to arterial elasticity and inversely proportional to peripheral vascular resistance. ND3 is the normalized value of D3. NA1 is the normalized ascending area, which is physiologically proportional to left ventricular systole and contractility, cardiac output, and blood pressure.

**Fig. 2:**
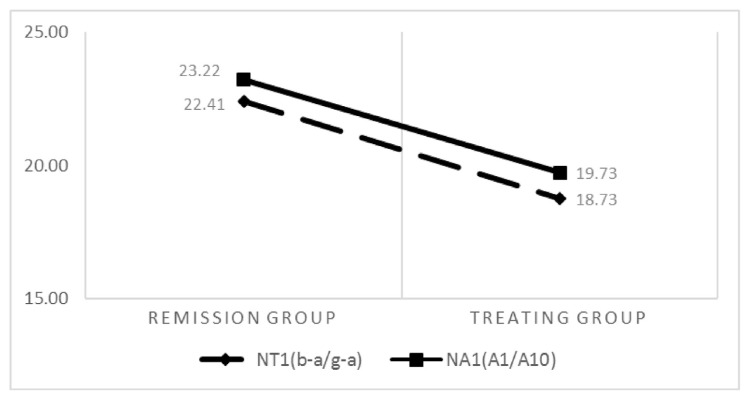
Comparison of NT1 and NA1 between the two groups.

**Fig. 3:**
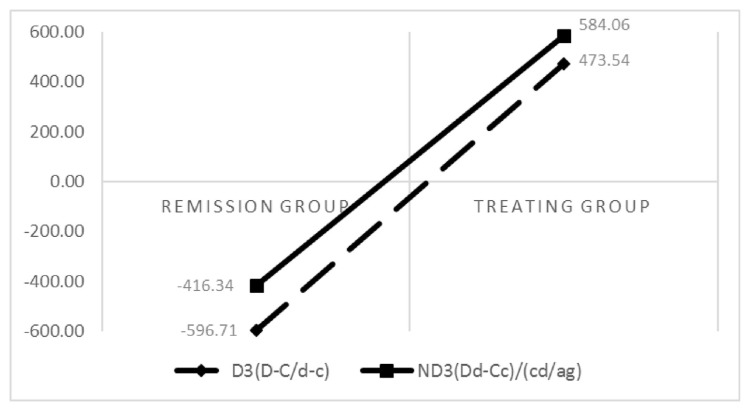
Comparison of D3 and ND3 between the two groups.

The comparison of the average values of D3 (D-C/d-c), ND3 (Dd-Cc)/(cd/ag), NA1 (A1/A10), and NT1 (b-a/g-a) in the treatment and remission groups revealed that NT1 and NA1 remission groups had higher values. Additionally, left ventricular contractility, cardiac output, and systolic blood pressure were stronger in the remission group. D3 and ND3 were higher in the treatment group than in the remission group, which indicates a worsening peripheral vascular resistance in the treatment group. In conclusion, cardiac output and peripheral resistance were poorer in the treatment group (Figs. 2 and 3).

## 4. Discussion

This study mainly used radial pulse waves to evaluate myocardial damage in patients with breast cancer. Chemotherapy causes heart damage, and Lin et al. found that chemotherapy further limits exercise capacity in patients with early-stage breast cancer. Basic information on various cardiovascular responses to exercise after chemotherapy in patients with early breast cancer [32]. Schoffel et al. also believe that chemotherapy can easily cause heart failure from the adverse effects of treatment. They compared the health care exposure and testing of patients with heart failure after breast cancer chemotherapy and cancer-free women and confirmed that chemotherapy is more likely to cause heart failure [33]. These two studies are consistent with the findings of this study that cardiac function was affected in patients during the treatment period. Echocardiography is the best method to detect chemotherapy-related heart damage. Mincu et al. used echocardiography to detect heart failure. The evaluation of left ventricular systolic heart failure is recognized as the key to treating patients; thus, Mincu specifically evaluates left ventricular diastolic dysfunction [34]. The pulse wave in this study was mainly used to evaluate the traditional left ventricular contractility. Fredslund et al. found that 1 year after breast cancer, microvascular dysfunction, measured by arterial waves, persisted without blood pressure changes or arterial stiffness [35]. Our findings revealed lower D3 and ND3 in the remission group, which indicates slow peripheral vascular resistance recovery. Unlike the study by Fredslund et al., it may be caused by the pulse wave measurement differences. Answatch, which was used in this study, has the function of measuring HRV and is also used by many TCM researchers to evaluate the HRV function [36,37]. This study mainly evaluates cardiovascular function; thus, HRV data was not used, which can be included in future prospective studies.

Among the parameters, NT1 was the rising slope of the pulse wave, which indicated the correlation between left ventricular contractility, cardiac output, and systolic blood pressure. Chest tightness, palpitations, and decreased blood pressure, which are common in patients with breast cancer who are receiving treatment, can be measured by NT1. D3 is the rising slope of the dicrotic wave, which means that the arterial elasticity increases and the peripheral vascular resistance decreases, that is, the relative resistance of cardiac output. Decreased cardiac output in patients with breast cancer who are receiving treatment may be related to increased resistance. NA1 is the normalized ascending area, which is physiologically proportional to left ventricular systole and contractility, cardiac output, and blood pressure. This value is similar to NT1. The radial pulse wave parameters found in this study can be used as markers for measuring myocardial disturbances in patients with breast cancer due to radiotherapy and chemotherapy. Through simple, non-invasive, and inexpensive pulse wave measurement, the side effects of treatment can be instantly understood, which can be of great help to the health of patients and is worthy of being used as an auxiliary test for breast cancer treatment. In our clinic, pulse wave examinations have been routinely provided every month to various patients with cancer. Additionally, this study is the first to find that pulse wave examinations are beneficial to patients with breast cancer. These four parameters will be further prospectively tracked, and similar benefits for other cancers are believed.

This study has a small sample size in the treatment group, and the continuous pulse wave data used are only four times; thus, the coefficient of variation is large, indicating that the data are more discrete. This may be because only four parameters can be detected from many parameters. However, the degree of dispersion has no difference between the two groups; thus, the comparison remained reliable. The chart review revealed no recorded cardiovascular-related symptoms in patients. The correlation between pulse waves and clinical symptoms is inadequate because medical records do not enforce the guidelines of the American Heart Association, which is especially for patients with breast cancer with potential cardiac risk. Therefore, the records of cardiovascular-related symptoms were incomplete; hence, they could not be uniformly included in the analysis. Therefore, future follow-up prospective studies are recommended to increase the sample size and document heart-related symptoms in detail.

## Figures and Tables

**Table 1:** Basic data of participants.

	Treatment group	Remission group
N	19	40
Gender	female	female
Age, year	49.11 ± 7.15	52.88 ± 9.26

**Table 2:** Comparison of CV for the treatment and remission group in time and frequency domains via t-test.

	Time domain	Frequency domain
N of parameters	45	50
*P*-value	0.091	0.049

**Table 3:** Comparison of the radial pulse wave in the treatment and remission group via t-test.

	Time domain			
parameter	D3 (D-C/d-c)	ND3 (Dd-Cc)/(cd/ag)	NA1 (A1/A10)	NT1 (b-a/g-a)
P	0.001*	0.014*	0.019*	0.023*
